# Long-COVID-19: the persisting imprint of SARS-CoV-2 infections on the innate immune system

**DOI:** 10.1038/s41392-023-01717-9

**Published:** 2023-12-14

**Authors:** Marianne Boes, Pascal Falter-Braun

**Affiliations:** 1 Center for Translational Immunology and Department of Pediatric Immunology, University Medical Center Utrecht, Utrecht University, Utrecht, The Netherlands; 2 Institute of Network Biology (INET), Molecular Targets and Therapeutics Center (MTTC), Helmholtz Munich, Neuherberg, Germany; 3 Microbe-Host Interactions, Faculty of Biology, Ludwig-Maximilians-Universität München, Planegg-Martinsried, Germany

**Keywords:** Innate immunity, Infection

In a recent *Cell* publication, Cheong et al. uncover how COVID-19 causes IL-6 induced epigenetic reprogramming of human immune stem cells, which causes lasting alterations in the composition and response characteristics of circulating immune cells.^1^ The study provides important insights into the mechanisms by which SARS-CoV-2 infections impact the human immune system and is an important hook into unraveling the mechanisms of post-acute sequelae of COVID-19 (PASC) commonly referred to as long-COVID.

While vaccination and drugs are reducing the societal impact of acute SARS-CoV-2 infections, between 10 and 40% of patients continue to suffer long after the acute infection has been cleared. The diverse PASC symptoms range from short breath and headaches to cognitive impairment (‘brain fog’) and debilitating fatigue. Not only are no treatments for PASC available but also the underlying molecular mechanisms remain opaque.^2^

Cheong et al. investigated in patients’ circulating immune cells if detectable changes persisted after clearance of the acute SARS-CoV-2 infection 3 weeks after the first symptoms. They assembled a cohort of COVID-19 convalescent patients, which was sampled between 1–3 and 4–12 months after SARS-CoV-2 infections requiring intensive care unit (ICU) admission and compared these patients to non-infected controls and to patients that had been on the ICU for different reasons. Focusing on peripheral blood mononuclear cells (PBMC) they investigated transcriptional or epigenetic changes using an integrated pipeline of single-nuclei transcriptome analysis and ATAC-seq sequencing, which identifies accessible chromatin regions. Among PBMCs CD14^+^ monocytes exhibited the most drastic changes. CD14^+^ monocytes are a group of heterogenous, short-lived antigen presenting cells that help orchestrating immune responses. Among these the authors could distinguish one cluster, M.SC3, which was more abundant even 12 months after the infection. Cells in this cluster resembled intermediate-type monocytes with functions that altogether resemble dendritic cells, the most effective amongst professional antigen presenting cells. In response to stimuli indicating viral infections, post-COVID monocytes showed up to 100-fold increased secretion of proinflammatory cytokines and enhanced transcriptional responses relating to cytokine signaling and monocyte activation. ATAC-seq also revealed a persistent pattern of differentially accessible chromatin which increased in abundance in early convalescent patients and did not return to the low levels observed in healthy individuals even 12 months after the acute infection. Thus, following severe SARS-CoV-2 infections, patients’ CD14^+^ monocytes carry specific and persistent epigenetic changes that puts them into an alerted state with heightened response characteristics.

Given that monocytes have a lifespan of a single day, the discovery of persistent epigenetic changes is notable and may reflect altered hematopoiesis and inheritance of epigenetic states from hematopoietic stem and progenitor cells (HSPC). To overcome the challenges associated with obtaining bone marrow resident HSPC, Cheong et al. developed a platform to enrich rare circulating HSPCs from PBMC and demonstrated that these faithfully represent the diversity and functional characteristics of their bone marrow-derived counterparts. With this platform, they discovered lasting epigenetic changes in HSPC of post-COVID patients that resembled those observed in mature monocytes. Especially late post-COVID HSPC exhibited skewed hematopoiesis with a significant increase of granulocyte monocyte precursor (GMP) cells. Intriguingly, the stem cells and the mature monocytes shared epigenetic signatures indicating that epigenetic and transcriptional programs are inherited by the mature progeny. The previously identified M.SC3 module activity was similarly increased in stem cells of the same patients.

Several molecular and cellular traits of post-COVID monocytes showed substantial variability among the patients, opening the question if intragroup variation can be related to post-COVID symptoms or other clinical features. A clear correlation of GMP variability was found to treatment with Tocilizumab, an antibody that blocks the interleukin 6 receptor (anti-IL6R). To demonstrate causal involvement of IL6 in the epigenetic reprogramming of HSPCs the authors turned to a mouse infection model, which reproduced the specific and persisting upregulation of GMP and the monocyte lineage after viral clearance. As in the clinical cohort, GMP and monocyte frequencies were reduced in the anti-IL6R treated mice, and molecular data revealed alterations of similar transcription factor activities and functional programs as in human cells. Importantly, the increased myelopoiesis and the poised inflammatory states of monocytes affect tissue recovery and inflammation. While naïve tissues were enriched for resident macrophages, lung tissue of recovered mice hosted more activated macrophages, accompanied by increased expression of genes mediating inflammation. The tissue level findings could be confirmed in lung autopsies of post-acute COVID-19 patients. Moreover, also in post-mortem brain tissue of the same patients a significant increase of monocytes was detectable. All these phenotypes were consistently milder in the anti-IL6R treated group.

The revelation that IL-6 induces epigenetic reprogramming of human immune stem cells, which changes the composition and response characteristics of circulating monocytes, is an important step towards understanding the etiology of PASC. Although, the study was underpowered to link the functional characteristics of post-COVID monocytes to PASC symptoms, the results provide a strong incentive for follow-up studies.

**Fig. 1:**
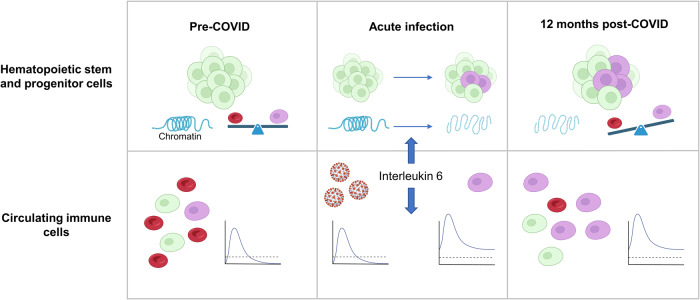
Interleukin 6 drives epigenetic reprogramming in hematopoietic stem and progenitor cells. In the pre-COVID state hematopoietic stem and progenitor cells (HSPC) cells generate balanced proportions of mature myeloid cells (top, left panel). IL6 levels during severe SARS-CoV-2 infections (middle panel) lead to epigenetic reprogramming of HSPCs giving rise to mature myeloid cells with heighted inflammatory response patterns (bottom). These epigenetic changes can persist 12 months after the initial infection and are accompanied by an altered composition of circulating immune cells in a heighted state of responsiveness (right). Artwork was created with BioRender.com

Beyond long-COVID, the strong data warrant appreciation for the role of IL6 in HSPC reprogramming. The persisting immune changes observed in post-COVID monocytes are reminiscent of similar persistent changes in diverse cell types that can be observed mostly after infections and have been coined ‘trained immunity’ or ‘innate immune memory’.^3^ The work by Cheong and colleagues is a major contribution towards understanding the underlying mechanisms. Beyond a possible general function of IL6 in establishing trained immunity, the PBMC-PIE platform will facilitate understanding stem cell reprogramming in diverse conditions and facilitate identification of additional factors that contribute to this important phenomenon. Lastly, the results from Cheong et al. may also shed new light on the mechanism of anti-IL6R therapies, which is an approved treatment for diseases ranging from rheumatoid arthritis to cytokine release syndrome associated with CAR-T-cell cancer therapy.^4^ As preventive targeting may often not be applicable it will be at least equally important to identify means to reprogram HSPC into an epigenetic state that has fewer debilitating consequences for the individual. The results by Cheong et al. constitute an important step towards understanding, preventing, and eventually treating the adverse consequences of an ill-trained innate immune system (Fig. 1).
